# Helicobacter pylori eradication for immune thrombocytopenia reduces non-response and raises platelet counts: Meta-analysis of randomized trials

**DOI:** 10.1007/s00277-026-07187-7

**Published:** 2026-07-28

**Authors:** Moath Hattab, Fathi Milhem, Hammam Jallad, Mahmoud Suleiman, Halla Albada, Ayman Khaled, Sakeena Saife, Mira Hallak, Sarah Saife, Fadi Abualhommos, Mohammad Bdair

**Affiliations:** Department of Medicine, Faculty of Medicine and Allied Medical Sciences, An-Najah National University, Nablus, P400 Palestine

**Keywords:** Immune thrombocytopenia, Helicobacter pylori, Platelet, Eradication therapy, Antibiotics, Immunology

## Abstract

Helicobacter pylori (H. pylori) has been implicated in immune thrombocytopenia (ITP), but the magnitude and reliability of benefit from eradication therapy in randomized trials remain uncertain. We performed a PRISMA-guided, PROSPERO-registered (CRD420251149478) systematic review and meta-analysis restricted to randomized controlled trials (RCTs) comparing H. pylori eradication versus control in ITP. Random-effects models generated risk ratios (RRs) for categorical outcomes: complete response (CR), partial response (PR), overall response (CR + PR), no/minimal response (NR), and 1-year relapse, and mean differences (MDs) for platelet-count change at approximately 6 months. Heterogeneity was assessed using I² and τ². Robustness was evaluated using leave-one-out analyses and additional post hoc sensitivity analyses for clinically or statistically influential trials. Small-study effects were explored using funnel plots and Egger/Begg tests when sufficient studies were available. Eight RCTs (*n* = 297) across Asia and Latin America met inclusion. Eradication increased CR without statistical significance (RR 1.45, 95% CI 0.87–2.41; I²=40.9%) and did not change PR (RR 0.88, 0.57–1.36; I²=0%) or overall response (RR 1.04, 0.90–1.20; I²=35.9%). NR was significantly lower with eradication (RR 0.65, 0.49–0.85; I²=14.8%). Relapse at 1 year trended lower but was not significant (RR 0.49, 0.24–1.01; I²=0%). Platelet counts rose more after eradication at ~ 6 months (MD + 61.9 × 10⁹/L, 36.1–87.7; I²=74.9%). Leave-one-out analyses did not materially change the overall conclusions. In a post hoc sensitivity analysis excluding Tang 2013 from the platelet-change outcome because of unusually small reported SDs, the platelet-count benefit remained significant and heterogeneity decreased to I²=0%. Funnel plots and formal tests suggested small-study effects for CR and overall response, indicating that these estimates may be inflated; no/minimal response showed no evidence of asymmetry. In randomized trials, H. pylori eradication in ITP was associated with a lower risk of no/minimal response and a greater platelet-count increase, while effects on CR, PR, overall response, and relapse remained uncertain. Because CR and overall response showed evidence of small-study effects, the most robust categorical finding was the reduction in no/minimal response. Larger, adequately powered trials are needed to define which patients are most likely to benefit and to confirm the durability of platelet response.

## Highlights (Clinical implications)

- H. pylori eradication was associated with a significant reduction in no/minimal response among patients with ITP.
- Eradication therapy was associated with a greater platelet-count increase at approximately 6 months, although heterogeneity was influenced by one trial.
- Effects on complete response, partial response, overall response, and relapse remain uncertain.
- Larger multicenter RCTs are needed to identify predictors of response and confirm long-term benefit.

## Introduction

Immune thrombocytopenia (ITP) is an acquired autoimmune disorder characterized by isolated thrombocytopenia (platelet count < 100 × 10^9^/L) due to accelerated platelet destruction and impaired platelet production, or sometimes secondary to other underlying conditions. First-line management for adults and children typically includes corticosteroids and, in selected scenarios, intravenous immunoglobulin (IVIG) or anti-D immunoglobulin for rapid, short-term increases in platelet count. Second-line options include thrombopoietin receptor agonists (TPO-RAs), rituximab, and splenectomy, each with important trade-offs related to durability of response, toxicity, cost, and patient preference [1, 2]. Despite multiple effective agents, relapses and treatment-related adverse effects are common, underscoring the need for safe, disease-modifying strategies.

Helicobacter pylori (H. pylori)–associated ITP was first noted when eradication therapy was followed by sustained platelet increases in a subset of patients. Proposed mechanisms include molecular mimicry and cross-reactive antibodies targeting platelet glycoproteins, which can drive platelet aggregation and immune-mediated clearance. Additional immune-modulatory pathways have been described, notably a shift in monocyte/macrophage Fcγ-receptor balance toward increased inhibitory FcγRIIB (relative to FcγRIIA) after eradication, correlating with reduced phagocytosis and platelet recovery. Overall, these mechanisms are biologically plausible but vary with host factors and bacterial strain virulence, and the strength of association appears to differ across regions with differing CagA-positive strain prevalence (higher in Eastern Asia vs. lower in Europe/North America) [3, 4].

Over the past two decades, numerous observational cohorts and several randomized controlled trials (RCTs) have evaluated whether H. pylori eradication improves platelet counts in ITP. A meta-analysis limited to RCTs suggested that eradication therapy confers a higher overall platelet response compared with control, although included trials were small and heterogeneous [5]. Pediatric RCTs also yield inconsistent results, with some showing no additional benefit of eradication to standard ITP management and others showing benefit for specific subgroups [6]. Long-term observational data suggest that if a response occurs, then it may be sustained after successful eradication but that generalizability to the wider non-high-prevalence setting is questionable [7].

Earlier syntheses combined RCTs with nonrandomized trials with heterogeneity in response definitions (e.g., complete response [CR], partial response), time of assessment, evidence of eradication (urea breath test vs. stool antigen vs. biopsy), and ancillary ITP therapy. Baseline platelet cutpoints, infection prevalence, and CagA status were also inconsistently documented, with difficulty of interpretation and external validity [3, 5]. These sources of heterogeneity, alongside limited blinding and small sample sizes, underscore the need for a focused, contemporary RCT-only evaluation with careful sensitivity analyses and qualitative assessment of clinical heterogeneity.

Therefore, this systematic review and meta-analysis aimed to evaluate the efficacy and safety of H. pylori eradication therapy compared with control in patients with H. pylori-positive ITP, using evidence restricted to randomized controlled trials. We assessed platelet-count change, CR, PR, overall response, no/minimal response, relapse, and adverse events where reported. Because prior trials differed in age group, geographic region, baseline platelet count, eradication confirmation, and background ITP therapy, these factors were considered important sources of clinical heterogeneity and were evaluated qualitatively when formal subgroup analysis was not feasible. The review followed PRISMA 2020 reporting guidance and was prospectively registered in PROSPERO (CRD420251149478).

## Methods

### Protocol & registration

This systematic review and meta-analysis was conducted in accordance with the Preferred Reporting Items for Systematic Reviews and Meta-Analyses (PRISMA) 2020 statement. The protocol was prospectively registered in the International Prospective Register of Systematic Reviews (PROSPERO; CRD420251149478) on 17 September 2025, before formal study selection and data extraction.

### Eligibility criteria

Eligible studies were randomized controlled trials evaluating H. pylori eradication therapy in patients with ITP and confirmed H. pylori infection. Participants of any age were eligible. H. pylori infection could be confirmed by urea breath test, stool antigen testing, rapid urease testing, histology/biopsy, or serology when accepted by the original trial protocol. Eligible comparators included placebo, observation/no eradication, or standard ITP care without eradication therapy. Studies were required to report at least one platelet-related outcome, including platelet-count change, CR, PR, overall response, no/minimal response, or relapse. Non-randomized studies, case reports, reviews, animal studies, studies without comparator arms, and studies without extractable platelet outcomes were excluded.

### Search strategy

We searched PubMed and Embase from database inception to September 2025 without language or date restrictions. The search strategy combined controlled vocabulary and free-text terms for H. pylori, immune thrombocytopenia, and eradication therapy. The PubMed search strategy was: (“Helicobacter pylori”[Mesh] OR “Helicobacter Infections”[Mesh] OR “Helicobacter pylori” OR “H. pylori” OR “Helicobacter Infection” OR “Helicobacter”) AND (“Purpura, Thrombocytopenic, Idiopathic”[Mesh] OR “Immune Thrombocytopenic Purpura” OR “ITP” OR “Idiopathic Thrombocytopenic Purpura” OR “Idiopathic Thrombocytopenia Purpura” OR “Immune Thrombocytopenia Purpura” OR “Immune Thrombocytopenia” OR “Idiopathic Thrombocytopenia”). Reference lists of eligible studies and relevant reviews were screened manually. We also searched conference proceedings, dissertation databases, and trial registries to identify additional published or unpublished data.

### Study selection (PRISMA flow diagram)

**Fig. 1:**
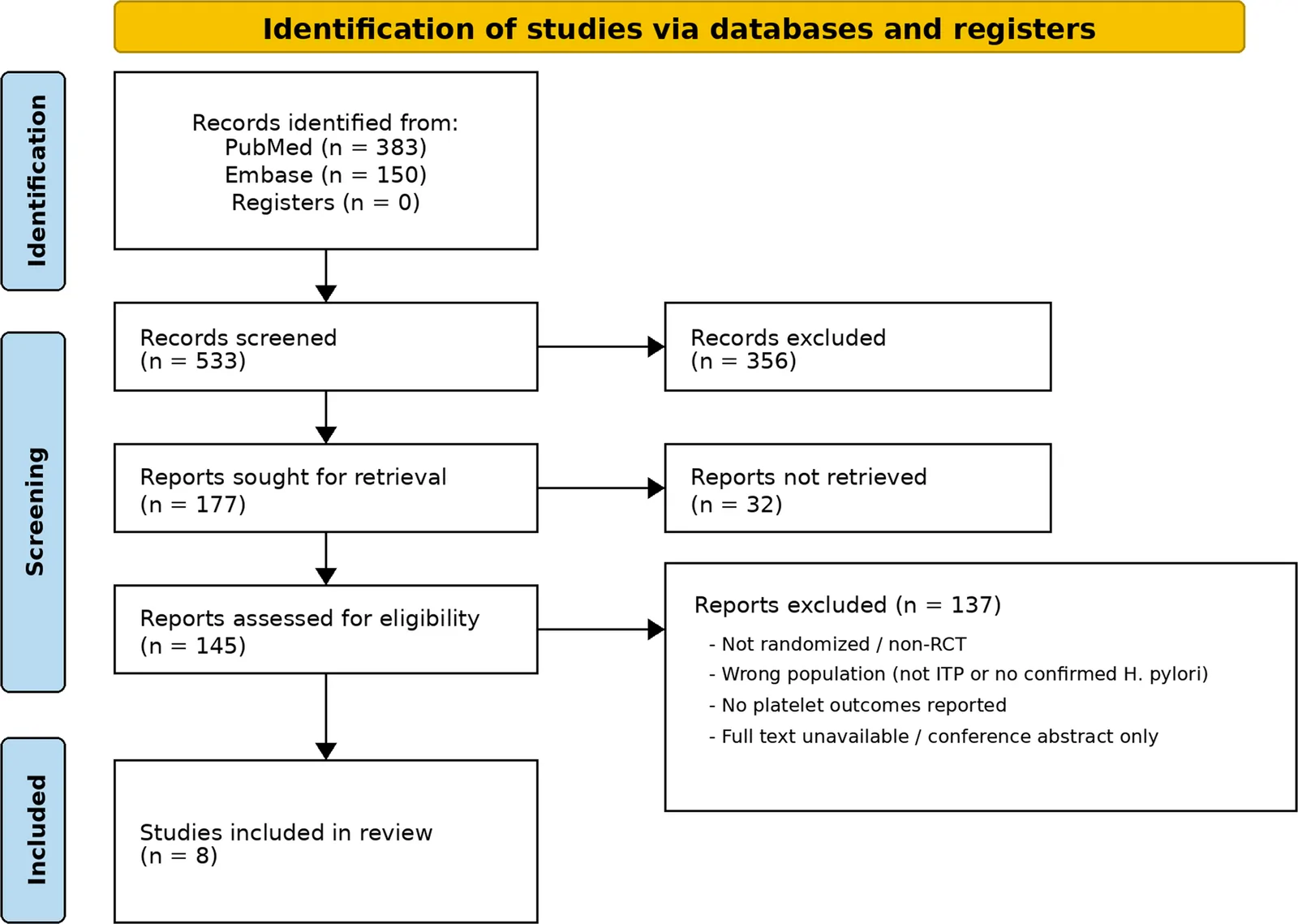
PRISMA flow diagram of study selection. The corrected flow distinguishes records screened, reports sought for retrieval, full-text reports assessed, and randomized trials included in the final synthesis

All identified records were imported into a reference manager and screened independently by two reviewers. Disagreements were resolved by discussion or third-reviewer adjudication. The database search identified 533 records, including 383 from PubMed and 150 from Embase. After title and abstract screening, 356 records were excluded as clearly irrelevant. The remaining 177 reports were sought for retrieval; 32 could not be retrieved, leaving 145 reports for full-text eligibility assessment. Of these, 137 reports were excluded because they were non-randomized studies, included an ineligible population, lacked confirmed H. pylori-positive ITP, had no comparator group, did not report platelet-related outcomes, or were conference abstracts without sufficient extractable data. Eight randomized controlled trials met the inclusion criteria and were included in the qualitative and quantitative synthesis. The study selection process is summarized in Fig. 1 [8].

### Data extraction and outcomes

Two investigators (H.J. and F.M.) independently extracted the variables of interest from each study. Disagreements were later resolved by discussion and consensus. The following were extracted from each study: first author, year of publication, country, total number of study participants, method of detection of H. pylori infection, number of patients with eradication given, number of patients without eradication given, duration of ITP, platelet count at enrollment and after 6 months, response, and relapse at 1 year.

Outcomes included platelet-count change at approximately 6 months (×10⁹/L), CR, PR, overall response (CR + PR), no/minimal response (NR), relapse at 1 year, and adverse events where reported.

### Data synthesis and statistical analysis

Study-level data were pooled using random-effects meta-analysis to account for expected between-study heterogeneity. For dichotomous outcomes, including CR, PR, overall response (CR + PR), no/minimal response, and relapse at 1 year, we calculated RRs with 95% CIs. For the continuous outcome of platelet-count change at approximately 6 months, we calculated MDs with 95% CIs, with platelet counts expressed as ×10⁹/L. When studies reported medians with interquartile ranges or ranges, values were converted to approximate means and SDs using established methods. Between-study heterogeneity was assessed using I² and τ², with I² values interpreted as low (< 30%), moderate (30–60%), or substantial (> 60%). Leave-one-out analyses were used to evaluate the influence of individual studies on pooled estimates.

Additional post hoc sensitivity analyses were performed in response to clinically or statistically important trial-level differences. Eghbali 2019 was examined separately in interpretation because it evaluated H. pylori eradication as an add-on to standard acute ITP therapy rather than as eradication versus observation alone. Tang 2013 was excluded from a sensitivity analysis of platelet-count change because the reported platelet SDs were unusually small for a clinical ITP cohort. Publication bias and small-study effects were assessed visually using funnel plots and, when enough studies were available, using Egger and Begg tests. Analyses were performed using R with the metafor package, with statistical significance set at *p* < 0.05.

### Risk of bias and quality assessment

**Fig. 2:**
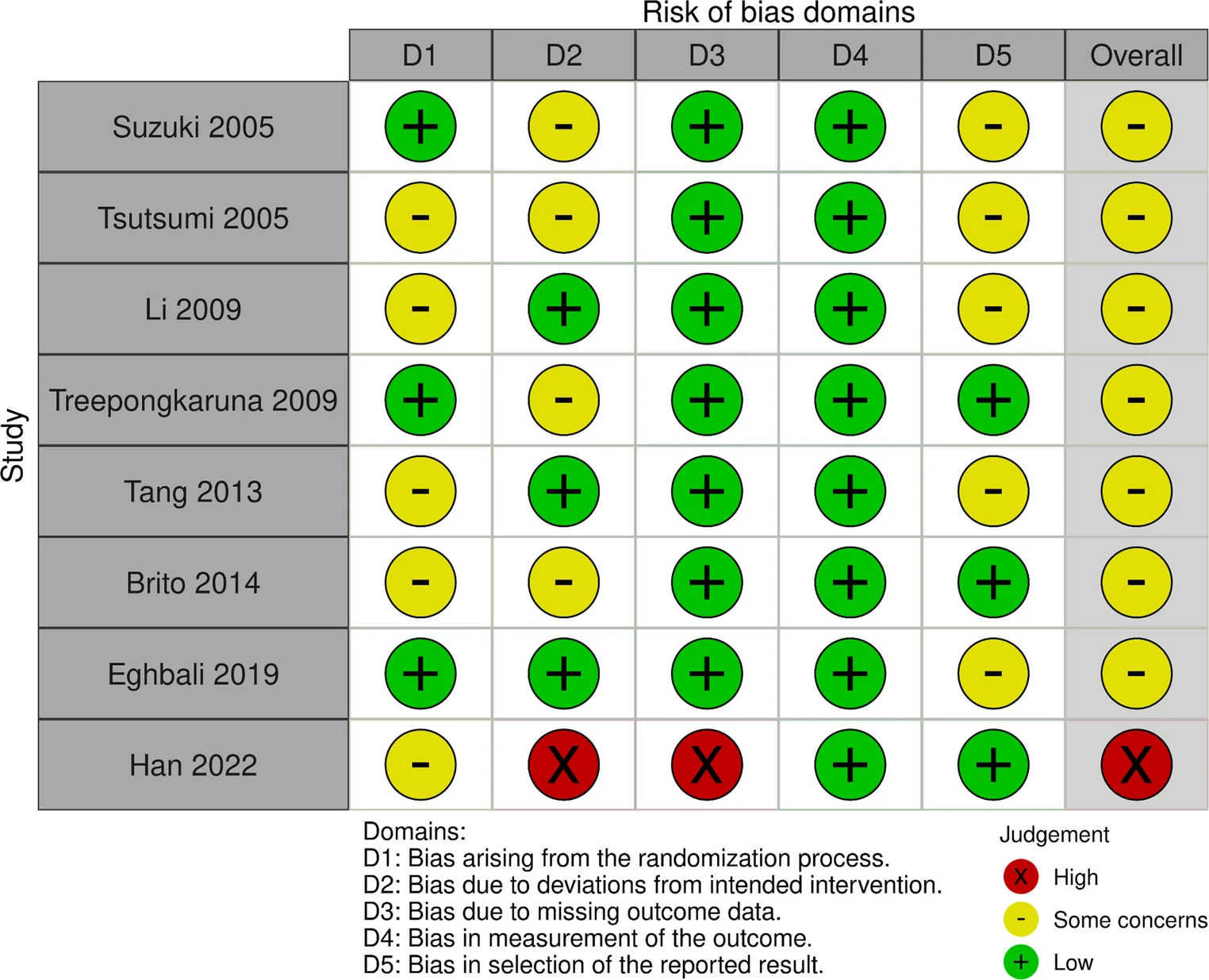
Traffic light plot of the risk of bias assessment for included randomized controlled trials using the Cochrane Risk of Bias 2 (RoB 2) tool. Each row represents an individual study, and each column corresponds to one of the five bias domains: randomization process, deviations from intended interventions, missing outcome data, measurement of the outcome, and selection of the reported result. Green indicates low risk of bias, yellow indicates some concerns, and red indicates high risk of bias

**Fig. 3:**
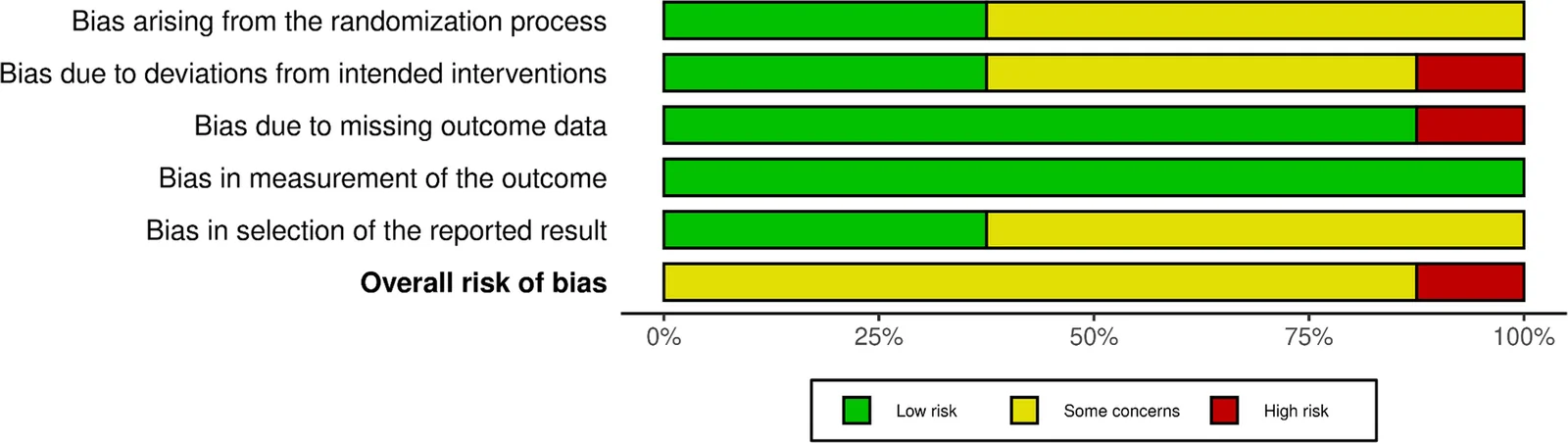
Summary plot of the risk of bias assessment. The figure displays the proportion of studies rated as low risk, some concerns, or high risk for each of the five domains

Risk of bias was assessed for all included randomized controlled trials using the Cochrane Risk of Bias 2 (RoB 2) tool. Each study was evaluated across five domains: the randomization process, deviations from intended interventions, missing outcome data, measurement of the outcome, and selection of the reported result. Two independent reviewers performed the assessments, with disagreements resolved through discussion or consultation with a third reviewer. The overall risk-of-bias judgments are summarized in Fig. 2, and domain-level assessments are presented in Fig. 3 [9, 10].

## Results

### Study characteristics

Eight randomized controlled trials involving 297 patients with ITP and confirmed H. pylori infection were included. Trials were published between 2005 and 2022 and were conducted in Japan, China, South Korea, Thailand, Iran, and Brazil. Several studies enrolled pediatric patients, whereas others enrolled adult patients with persistent or chronic ITP. Control arms varied across trials and included observation, PPI monotherapy, prednisone/prednisolone, or standard ITP therapy without eradication. Table 1 summarizes the baseline characteristics, diagnostic methods, interventions, and outcomes of the included trials.

### Subgroup and heterogeneity considerations

Quantitative subgroup analyses by age group, geographic region, baseline platelet count, and eradication success were not performed because only eight small RCTs were included and these variables were inconsistently reported. Several trials enrolled exclusively pediatric or adult populations, baseline platelet thresholds differed across studies, eradication success was reported using different denominators, and individual-patient data were unavailable. These factors were therefore considered qualitatively when interpreting heterogeneity and external validity.

### Patient characteristics

### Diagnostic methods and treatment combinations

H. pylori diagnostic methods varied across trials. UBT was the most frequently used test, either alone or with histology or stool antigen testing. Suzuki 2005 used UBT or histology; Tsutsumi 2005 used anti-H. pylori antibody testing; Li 2009 and Treepongkaruna 2009 used UBT; Tang 2013 used ¹³C-UBT; Brito 2014 used UBT or stool antigen testing; Eghbali 2019 used stool antigen testing; and Han 2022 accepted UBT, rapid urease testing, stool antigen testing, or biopsy through gastroscopy. Eradication confirmation was also variably reported, including stool antigen testing in Eghbali 2019 and UBT at 3 months in Han 2022.

Eradication regimens were mostly PPI-based triple therapies, commonly including amoxicillin and clarithromycin, with some variation in duration and co-interventions. Several pediatric trials used concomitant prednisone/prednisolone in both trial arms, while Eghbali 2019 evaluated eradication as an add-on to standard acute ITP therapy. Han 2022 evaluated sequential eradication therapy in adults with moderate thrombocytopenia. Because background ITP therapy and control-arm design differed across trials, these differences were considered when interpreting pooled estimates (Table 1; [6, 11–17]).

**Table 1:** Baseline characteristics and outcomes of included randomized controlled trials evaluating H. pylori eradication in ITP

First author (yr), country [ref]	ITP + Hp pts	Hp Dx	Arm	*n*	Age, yr	ITP dur, yr	PLT enr	PLT 6 mo	ΔPLT 6 mo	Resp* (CR + PR)	Relapse 1 yr
Suzuki (2005), Japan [11]	25	UBT / hist.	ERD	13	57.4 ± 15.0	5.8 ± 7.2	54.7 ± 26.9	114.5 ± 90.5	59.8 ± 80.5	6 (46.2%)	NA
	25	UBT / hist.	Obs	12	56.2 ± 7.8	4.6 ± 5.2	48.4 ± 22.1	48.1 ± 26.0	−0.3 ± 24.3	0 (0%)	NA
Tsutsumi (2005), Japan [12]	17	anti-Hp Ab	ERD	9	60.3 ± 9.85	NA	5.38 ± 0.99	NA	NA	6 (66.7%)	2 (33.3%)
	17	anti-Hp Ab	PPI	8	63.3 ± 12.56	NA	5.38 ± 1.59	NA	NA	5 (62.5%)	2 (40%)
Li (2009), China [13]	93	UBT	ERD + PD	51	6.7 ± 2.4	NA	NA	NA	NA	45 (88.2%)	11 (21.6%)
	93	UBT	PD	42	5.8 ± 2.7	NA	NA	NA	NA	37 (88.1%)	17 (40.5%)
Treepongkaruna (2009), Thailand [14]	16	UBT	ERD + PD	7	10.88 ± 1.93	3.85 ± 1.5	33.25 ± 29.68	NA	NA	1 (14.3%)	NA
	16	UBT	PD	9	11.4 ± 2.6	5.23 ± 2.08	39.25 ± 27.78	NA	NA	1 (12.5%)	NA
Tang (2013), China [15]	68	¹³C-UBT	ERD + PD	34	NA	NA	14.8 ± 0.4	160.4 ± 1.0	145.6 ± 0.9	26 (76.5%)	6 (17.6%)
	68	¹³C-UBT	PD	34	NA	NA	15.1 ± 0.3	80.6 ± 1.1	65.5 ± 0.98	20 (58.8%)	16 (47.1%)
Brito (2014), Brazil [16]	22	UBT / stool Ag	ERD ± PD	11	12.0 ± 4.0	4.8 ± 2.2	54.0 ± 45.2	128 ± 73	74.0 ± 63.8	7 (63.6%)	NA
	22	UBT / stool Ag	No ERD ± PD	11	11.1 ± 3.7	4.4 ± 3.2	60.3 ± 41.1	63 ± 44	2.7 ± 42.6	4 (36.4%)	NA
Eghbali (2019), Iran [6]	28	HpSA	Std ITP + ERD	14	6.3 ± 1.3	NA	16.93 ± 10.1	175 ± 70.8	158.1 ± 66.3	14 (100%)	NA
	28	HpSA	Std ITP	14	5.9 ± 1.1	NA	15.0 ± 3.8	150 ± 55.3	135 ± 53.5	14 (100%)	NA
Han (2022), South Korea [17]	28	UBT / RUT / stool Ag / Bx	ERD	17	48.5 ± 14.5	5.7 ± 5.1	54.25 ± 3.74	122.7 ± 26.79	68.45 ± 25.1	9/15 (60.0%)†	NA
	28	UBT / RUT / stool Ag / Bx	Obs	11	51 ± 18.2	8.1 ± 8.2	56.13 ± 8.25	72.33 ± 20.63	16.2 ± 17.99	0/7 (0%)†	NA

*Abbreviations: Hp *H. pylori, *Dx* diagnosis, *pts *patients, *ERD* eradication therapy, *Obs *observation/no eradication, *Std ITP* standard ITP therapy, *PD* prednisone/prednisolone, *PPI *proton-pump inhibitor, *UBT* urea breath test, *RUT *rapid urease test, *Bx* biopsy, *HpSA *H. pylori stool antigen, *PLT *platelet count, *enr *enrollment, *CR *complete response, *PR *partial response, *NA *not available

* Response definitions varied across trials and followed each study’s original criteria

† For Han 2022, response is shown according to the primary endpoint population: successfully eradicated patients in the treatment arm versus evaluable controls at 3 months

Tang 2013 reported unusually small platelet-count SDs; values were extracted as reported and examined in sensitivity analysis

For Eghbali 2019, both groups received standard ITP therapy; therefore, the comparison reflects the incremental effect of adding eradication therapy rather than eradication versus observation alone

### Response, adverse events, sensitivity analyses, and publication bias

**Fig. 4:**
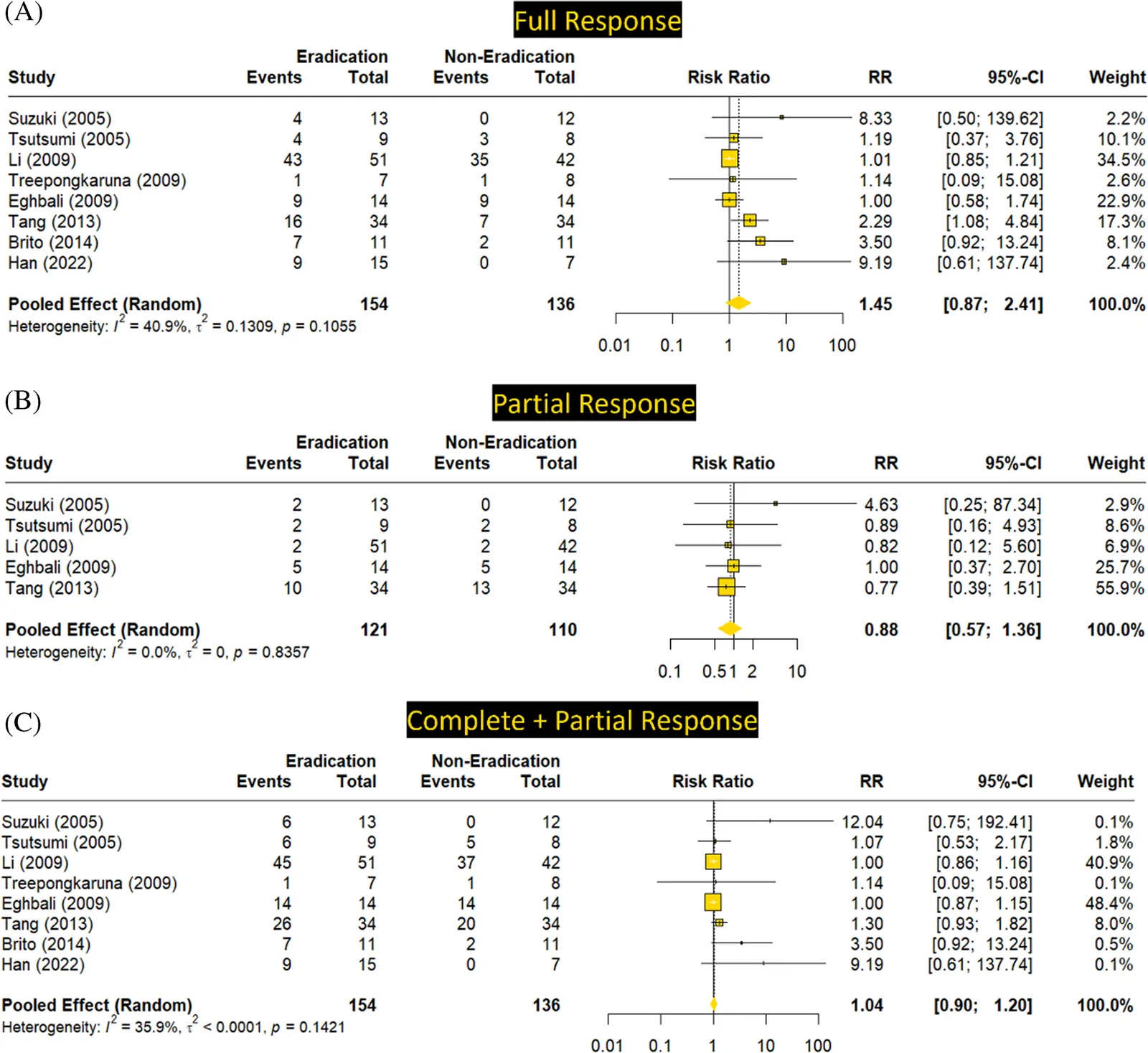
Meta-analysis forest plots, part 1: complete response (**A**), partial response (**B**), and overall response (**C**) after H. pylori eradication versus control in ITP randomized trials. Markers show study RRs with 95% CIs; diamonds show random-effects pooled estimates

**Fig. 5:**
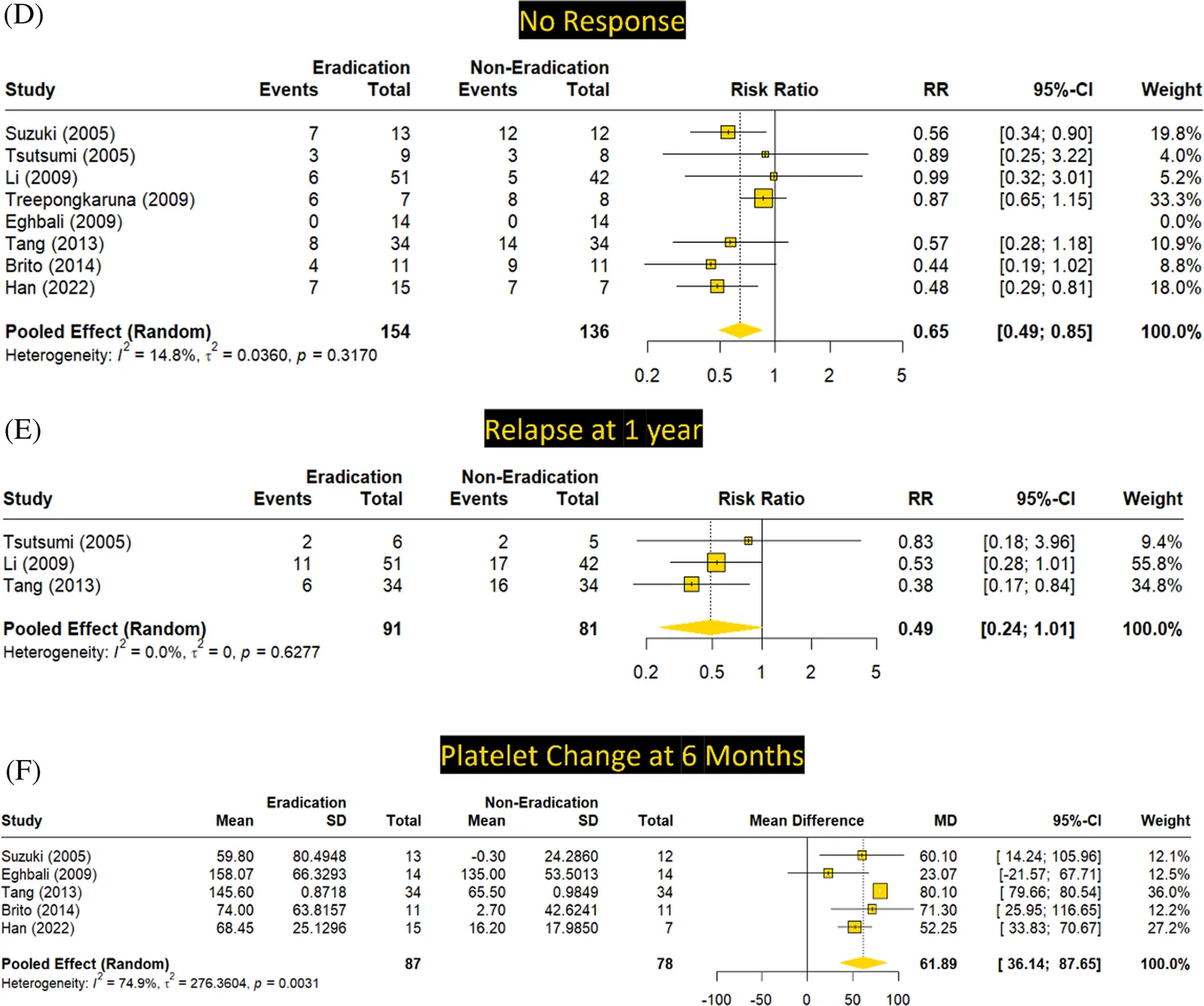
Meta-analysis forest plots, part 2: no/minimal response (**D**), relapse at 1 year (**E**), and platelet-count change at approximately 6 months (**F**; MD, ×10⁹/L)

Across randomized trials, CR favored eradication but was not statistically significant (RR 1.45, 95% CI 0.87–2.41; I²=40.9%, τ²=0.1309; Q *p* = 0.106; Fig. 4A). PR did not differ between groups (RR 0.88, 95% CI 0.57–1.36; I²=0%, τ²=0; Q *p* = 0.836; Fig. 4B). When CR and PR were combined as overall response, the pooled effect remained neutral (RR 1.04, 95% CI 0.90–1.20; I²=35.9%, τ²<0.0001; Q *p* = 0.142; Fig. 4C). By contrast, the risk of no/minimal response was significantly lower with eradication (RR 0.65, 95% CI 0.49–0.85; I²=14.8%, τ²=0.0360; Q *p* = 0.317; Fig. 5D). At 1 year, relapse trended lower after eradication without reaching statistical significance (RR 0.49, 95% CI 0.24–1.01; I²=0%, τ²=0; Q *p* = 0.628; Fig. 5E). For platelet-count change at approximately 6 months, eradication was associated with a greater increase versus control (MD + 61.89 × 10⁹/L, 95% CI + 36.14 to + 87.65; I²=74.9%, τ²=276.36; Q *p* = 0.003; Fig. 5F), indicating substantial between-study heterogeneity.

**Fig. 6:**
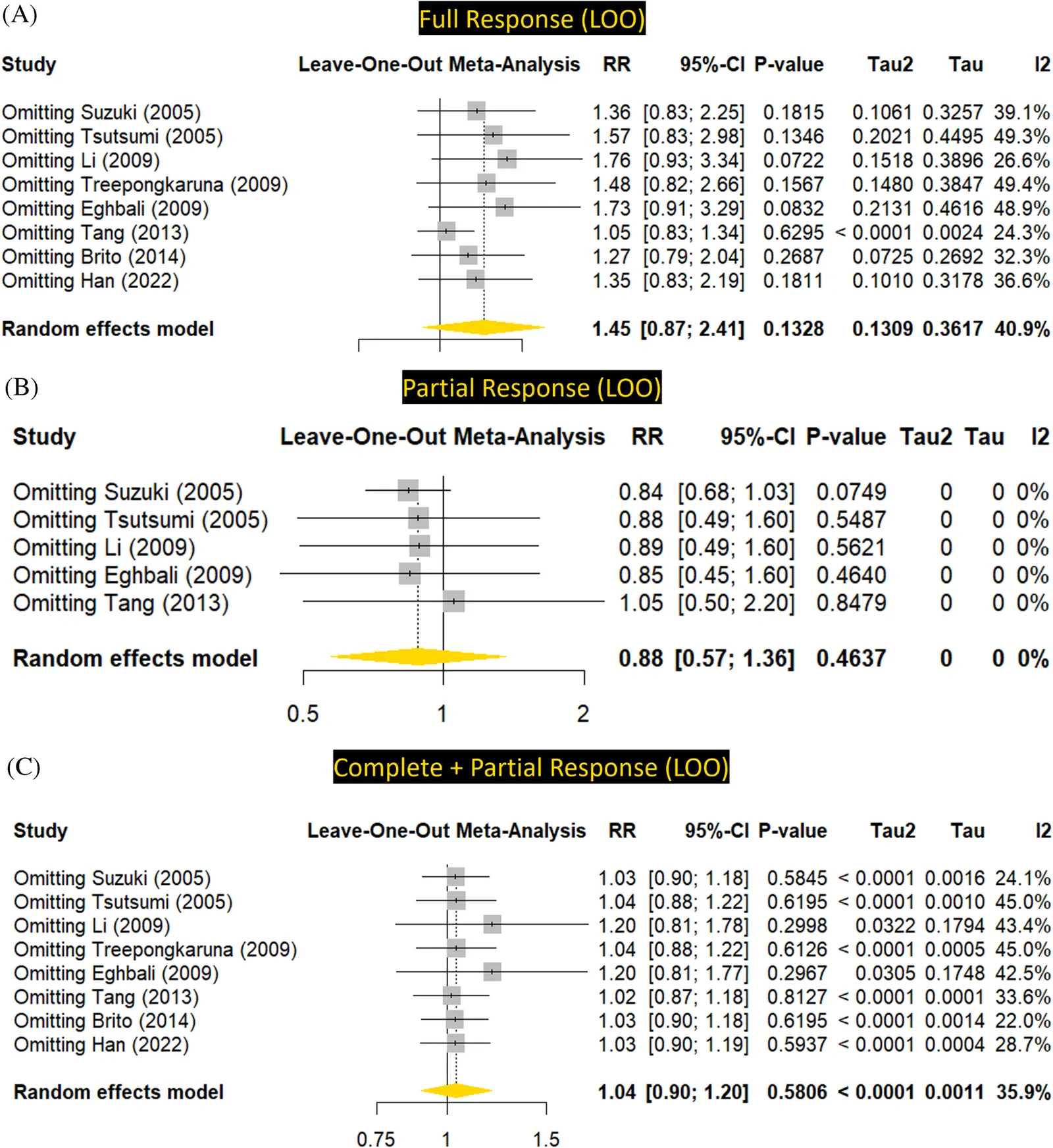
Leave-one-out sensitivity analyses, part 1: complete response (**A**), partial response (**B**), and overall response (**C**). Pooled effects were re-estimated after omitting each study in turn

**Fig. 7:**
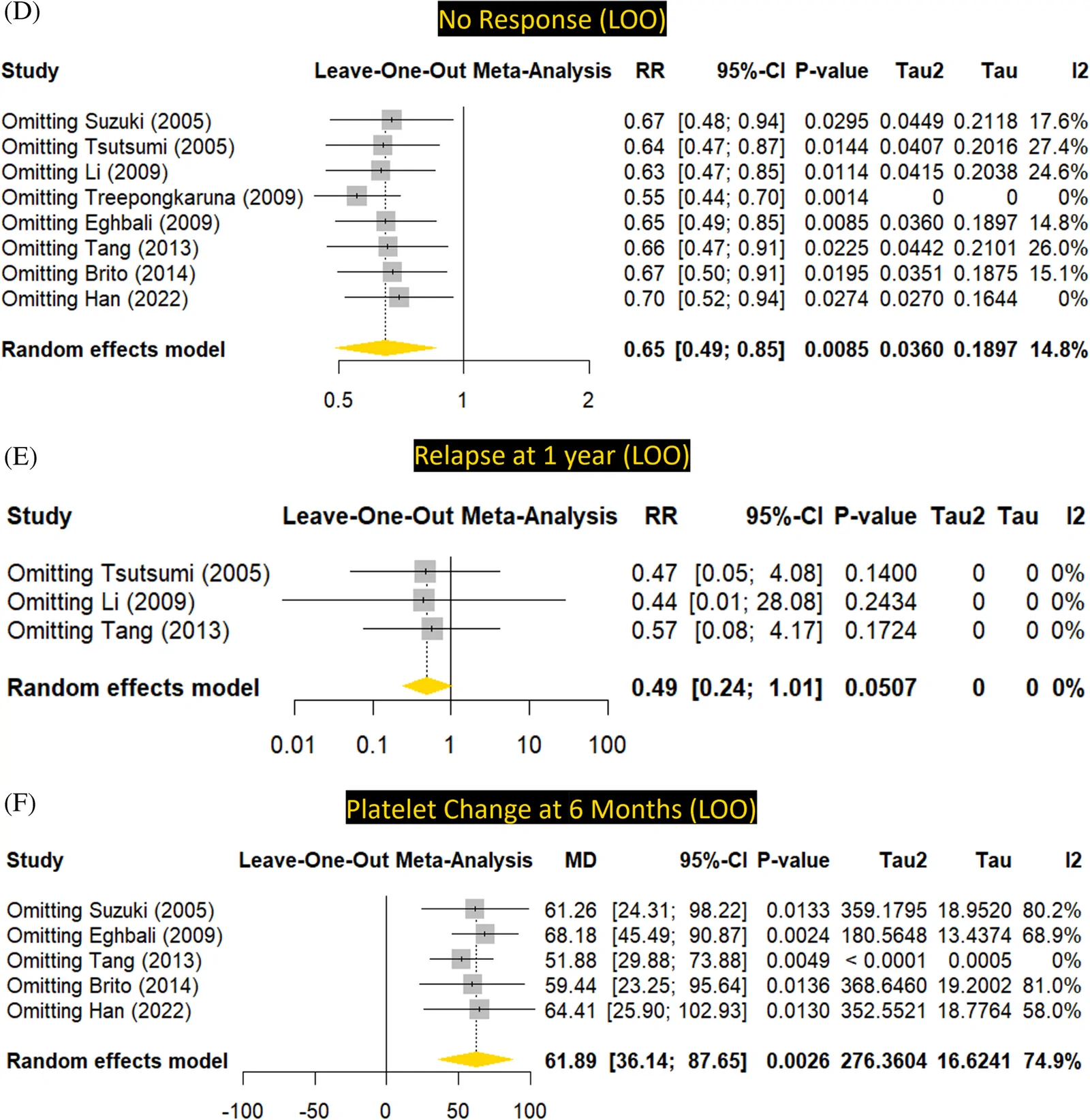
Leave-one-out sensitivity analyses, part 2: no/minimal response (**D**), relapse at 1 year (**E**), and platelet-count change at approximately 6 months (**F**; MD, ×10⁹/L)

Leave-one-out analyses did not materially alter the direction of the main conclusions (Figs. 6 and 7). However, Tang 2013 was the main contributor to heterogeneity in platelet-count change. (Table 1; [15]). Because Tang 2013 reported unusually narrow platelet-count SDs and a larger platelet increase than other trials, a post hoc sensitivity analysis was performed excluding this study from the platelet-change outcome. After exclusion of Tang 2013, the platelet-count benefit remained significant while heterogeneity resolved (MD + 51.9 × 10⁹/L, 95% CI + 36.8 to + 66.9; I²=0%). This suggests that Tang 2013 accounted for most of the heterogeneity in platelet-count change, while the direction and significance of the platelet benefit were preserved.

Eghbali 2019 was retained in the primary analysis because both randomized arms received standard ITP therapy, allowing the trial to estimate the incremental effect of adding eradication therapy (Table 1; [6]). However, its design differs from trials comparing eradication with observation or placebo, and this distinction was considered in sensitivity interpretation. Adverse events were variably and sparsely reported across trials and were not meta-analyzed. Available reports generally described mild gastrointestinal symptoms, with no unexpected safety signal; however, certainty around harms remains limited.

**Fig. 8:**
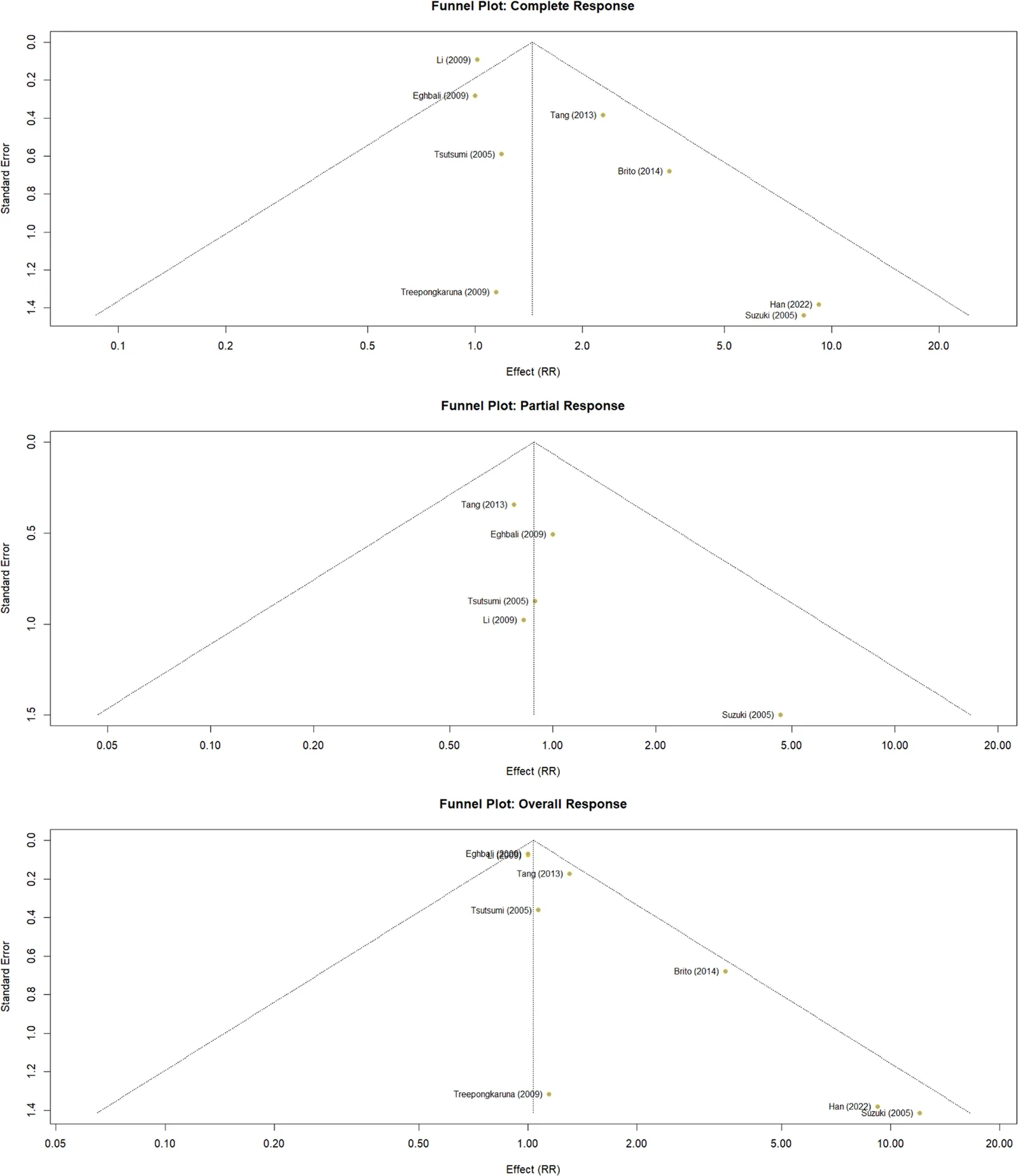
Funnel plots for small-study effects, part 1: complete response, partial response, and overall response

**Fig. 9:**
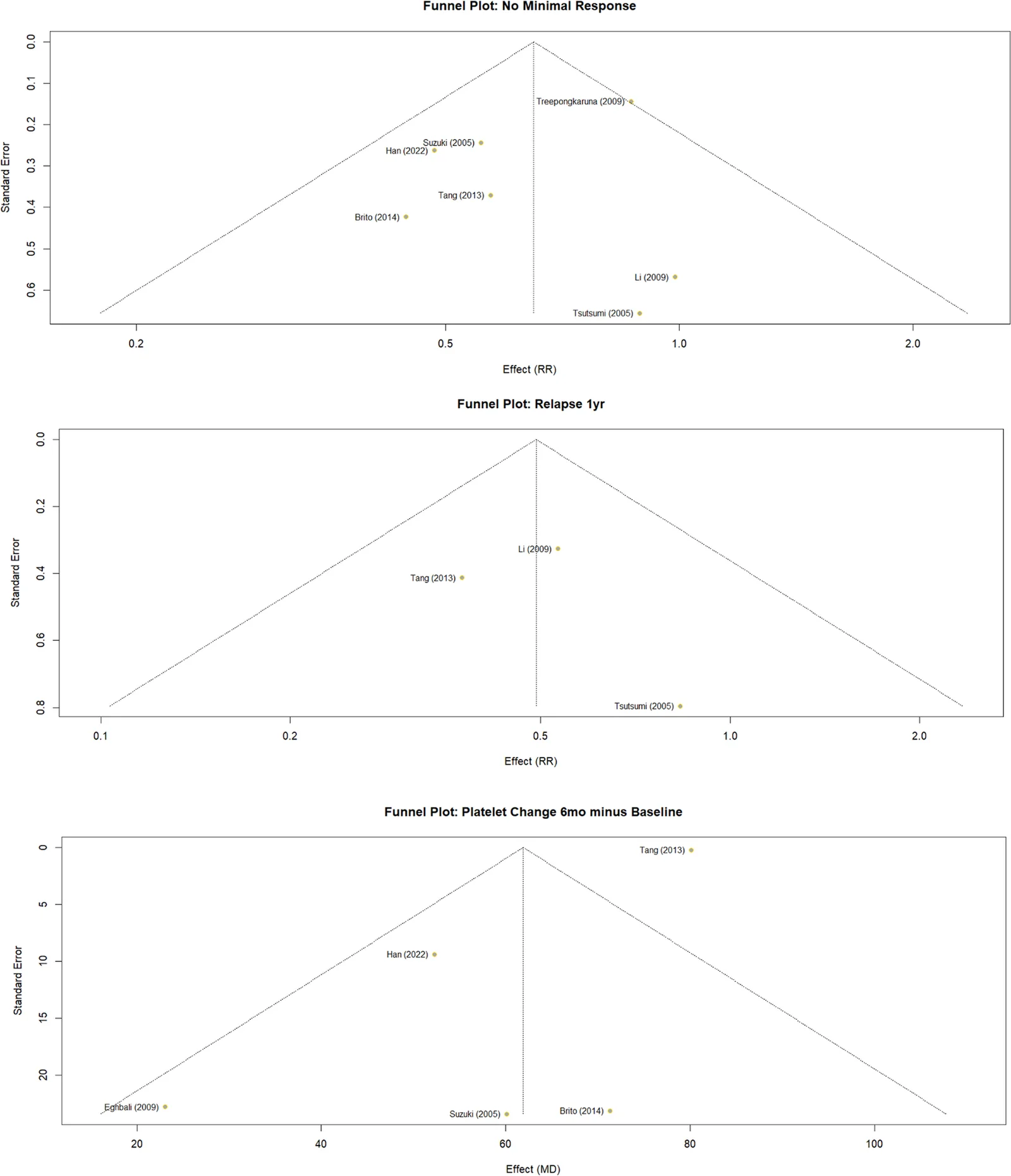
Funnel plots for small-study effects, part 2: no/minimal response, relapse at 1 year, and platelet-count change at approximately 6 months

Visual inspection of funnel plots suggested asymmetry for CR and overall response, supported by formal testing (Egger *p* = 0.024 for CR; Egger *p* = 0.012 and Begg *p* = 0.048 for overall response; Fig. 8). These findings suggest that the CR and overall response estimates may be inflated by small-study effects or selective publication. In contrast, no/minimal response showed no evidence of asymmetry (Egger *p* = 0.408; Begg *p* = 0.881; Fig. 9). PR showed no significant evidence of asymmetry, although the number of studies was small (Fig. 8). Relapse at 1 year included too few studies for reliable publication-bias assessment (Fig. 9). For platelet-count change, Egger’s test was borderline (*p* = 0.077) and Begg’s test was null (*p* = 1.000), with interpretation limited by the small number of studies and the influence of Tang 2013 (Fig. 9).

Taken together, randomized evidence suggests that H. pylori eradication reduces the proportion of patients with no/minimal response and increases platelet counts at approximately 6 months. However, effects on CR, PR, overall response, and relapse are less certain because of small sample sizes, heterogeneous response definitions, variable background ITP therapy, and evidence of small-study effects for CR and overall response.

## Discussion

This randomized-trial-only meta-analysis suggests that H. pylori eradication in immune thrombocytopenia is associated with two main findings: a lower risk of no/minimal response and a greater platelet-count increase at approximately 6 months. In contrast, pooled effects on complete response, partial response, and overall response were not statistically significant, and the apparent directional benefit for complete response and overall response should be interpreted cautiously because funnel-plot asymmetry and Egger testing suggested possible small-study effects. Therefore, the most robust categorical finding in this review is the reduction in no/minimal response.

The reduction in no/minimal response is clinically relevant because it suggests that eradication therapy may reduce the proportion of patients who fail to achieve any meaningful platelet improvement. This finding was statistically significant, showed low heterogeneity, and did not demonstrate evidence of funnel-plot asymmetry. In contrast, complete response and overall response were more vulnerable to uncertainty because response definitions varied across trials, sample sizes were small, and publication-bias testing suggested that these estimates may be inflated. Taken together, the categorical outcomes support a cautious interpretation: eradication therapy may improve the likelihood of achieving some platelet benefit, but the probability of achieving complete or overall response remains uncertain.

The platelet-count change outcome also supports a potential benefit of eradication therapy, but its magnitude requires careful interpretation. In the primary analysis, eradication was associated with a significant platelet-count increase at approximately 6 months, but heterogeneity was substantial. Tang 2013 reported unusually small platelet-count SDs and a larger platelet rise than other trials [15]. When Tang 2013 was excluded in a post hoc sensitivity analysis, the platelet-count benefit remained significant and heterogeneity decreased to I²=0%. This suggests that the direction of effect is robust, but the pooled magnitude of platelet improvement may be influenced by this trial and should not be overinterpreted.

Differences in trial design also affected interpretation. Eghbali 2019 evaluated eradication as an add-on to standard acute ITP therapy rather than against observation alone [6]. This trial was retained because randomization preserved a matched standard-treatment background, allowing estimation of the incremental effect of eradication therapy. However, its acute pediatric population and active-control design limit direct comparability with trials evaluating eradication against observation, placebo, or no eradication [6, 11, 12, 14, 16, 17].

Several biologically plausible mechanisms may explain the association between H. pylori infection and ITP. The most frequently proposed pathway is molecular mimicry, particularly involving immune responses to CagA-positive H. pylori strains that may cross-react with platelet glycoproteins and promote immune-mediated platelet clearance [3, 4, 18]. Eradication may also modify monocyte/macrophage Fcγ-receptor balance and reduce platelet phagocytosis [4, 19]. These mechanisms provide biological plausibility for platelet recovery after eradication, but they remain incompletely defined and may vary by geography, bacterial strain, host immune background, and baseline ITP phenotype [3, 4]. Mechanistic data should therefore be interpreted as supportive rather than definitive.

Geographic and clinical heterogeneity may also explain why responses to eradication therapy differ across studies. Prior literature has suggested that platelet responses after eradication may be more common in regions with higher H. pylori prevalence or different bacterial virulence patterns [3, 4, 7]. The trials included in this review were conducted across Asia and Latin America, enrolled both adult and pediatric populations, and differed in baseline platelet thresholds, ITP chronicity, eradication regimens, and background ITP therapy [6, 11–17]. These differences limit the generalizability of a single pooled estimate and suggest that eradication therapy may not have a uniform effect across all ITP populations.

The publication-bias assessment further supports a cautious interpretation. Complete response and overall response showed funnel-plot asymmetry with significant Egger tests, suggesting that these estimates may be inflated by small-study effects or selective publication. In contrast, no/minimal response showed no evidence of funnel asymmetry and had low heterogeneity, making it the most internally consistent categorical outcome in this review. The platelet-count change analysis also requires caution because the number of studies was small and one trial contributed substantially to heterogeneity [15].

Overall, these findings suggest that H. pylori testing and eradication may be reasonable in patients with confirmed H. pylori-positive ITP, particularly in settings where H. pylori infection is common and eradication therapy is accessible, inexpensive, and generally safe [1–4]. However, the evidence does not support overstating eradication as a uniformly effective ITP treatment. Rather, eradication appears most likely to reduce the probability of no/minimal platelet response and may increase platelet counts in selected patients. Larger multicenter randomized trials using standardized response definitions, consistent eradication confirmation, and longer follow-up are needed to identify predictors of response and clarify durability.

## Limitations

This review has several limitations. First, only eight randomized controlled trials with small sample sizes were available, limiting statistical power and precision [6, 11–17]. Second, response definitions, follow-up timing, background ITP therapy, and control-arm design varied across trials [6, 11–17]. Third, quantitative subgroup analyses by age group, geographic region, baseline platelet count, and eradication success were not reliable because of the small number of studies, inconsistent aggregate reporting, and absence of individual-patient data. These variables were therefore considered qualitatively rather than formally pooled as subgroups.

Fourth, Eghbali 2019 evaluated eradication as an add-on to standard acute ITP therapy, limiting direct comparability with observation-controlled trials [6]. Fifth, Tang 2013 reported unusually small platelet-count SDs and accounted for most of the heterogeneity in platelet-count change [15]. Although sensitivity analysis excluding Tang 2013 preserved the platelet-count benefit and reduced heterogeneity to I²=0%, the exact pooled magnitude of platelet improvement should be interpreted cautiously. Sixth, adverse events were inconsistently reported and could not be meta-analyzed, limiting certainty regarding harms. Finally, funnel-plot asymmetry for complete response and overall response suggests possible small-study effects or selective publication, so these outcomes should be interpreted with caution.

## Conclusion

In randomized trials, H. pylori eradication in ITP was associated with a reduced risk of no/minimal response and a greater platelet-count increase at approximately 6 months. Effects on complete response, partial response, overall response, and relapse remain uncertain, particularly because complete response and overall response showed evidence of small-study effects. Given the low cost and generally favorable safety profile of eradication therapy, testing for and treating confirmed H. pylori infection may be reasonable, especially in high-prevalence settings. Larger multicenter randomized trials are needed to identify predictors of response, confirm durability, standardize outcome definitions, and clarify which patients are most likely to benefit.

## Data Availability

The extracted data and analysis code are available from the corresponding author upon reasonable request.

## Declarations

### Protocol and registration

The protocol was prospectively registered in PROSPERO (CRD420251149478). Reporting follows PRISMA 2020.
